# Gatekeepers of Pain: A Scoping Review on How Nanoparticle Engineering Illuminates Selective Transient Receptor Potential Vanilloid 1 (TRPV1) Targeting

**DOI:** 10.7759/cureus.107610

**Published:** 2026-04-23

**Authors:** Alexa Q Xiang, Emily Cho, Tiffany Jiang, Nikita Balraj, Shadi Abu-Baker

**Affiliations:** 1 Academy Program, The Ohio State University, Columbus, USA; 2 Medicine, Washington High School, Fremont, USA; 3 Medicine, N.Y.C. Lab School for Collaborative Studies, New York City, USA; 4 Medicine, Dougherty Valley High School, San Ramon, USA; 5 Chemistry and Biochemistry, Ohio University, Zanesville, USA

**Keywords:** chronic pain, nanoparticles, pharmaceutical chemistry, targeted therapeutics, transient receptor potential vanilloid 1 (trpv1)

## Abstract

Conventional analgesics often provide limited relief for chronic pain and can cause systemic side effects. This scoping review aims to analyze mechanistic and bibliometric trends in nanoparticle-engineered delivery systems designed to selectively modulate transient receptor potential vanilloid 1 (TRPV1) receptors for precision chronic pain therapy; following PRISMA 2018 guidelines, the first 100 top-cited records from the Web of Science (WoS) Core Collection were organized in Microsoft Excel (Microsoft® Corp., Redmond, WA) and BibTeX (Oren Patashnik, Stanford University, Stanford, CA) for bibliometric analysis, with data also being qualitatively synthesized. Citation patterns were concentrated among a few leading researchers and institutions, highlighting the value of aligning with established funding bodies. Advanced polymeric and magnetic nanoparticles demonstrated the ability to cross the blood-brain barrier and selectively modulate TRPV1-mediated pain pathways. Nanoparticles carrying charged capsaicinoids improved bioavailability and reduced neuroinflammation relative to free capsaicin. Dose-dependent effects were consistently observed, as sustained low-dose release produced receptor desensitization and analgesia, while burst or high-dose delivery caused neuronal ablation. Surface-functionalized nanoparticles, particularly those with TRPV1-binding ligands or redox-responsive coatings, enhanced receptor specificity and reduced transient receptor potential ankyrin 1 (TRPA1) co-activation. Rationally engineered nanoparticles optimized for size, charge, ligand density, and release kinetics present a promising avenue for safer, more effective chronic pain therapies. By selectively modulating TRPV1 while mitigating thermoregulatory disruption, researchers can achieve long-lasting analgesia by prioritizing targeting precision to advance sustainable chronic pain treatments.

## Introduction and background

Chronic pain is a major and persistent global health challenge, affecting a large proportion of the adult population and contributing to disability and reduced quality of life that prompts increased healthcare utilization. Despite decades of pharmacological development, chronic pain management remains difficult because many commonly used analgesics, medications that reduce pain, provide only partial relief and are associated with adverse long-term side effects. These limitations have driven increasing interest in mechanism-based pain therapies that target specific receptor pathways, instead of broadly suppressing neural activity throughout the nervous system.

One such receptor, the transient receptor potential vanilloid 1 (TRPV1) ion channel, has been extensively studied as a molecular target in pain biology. In simple terms, TRPV1 is a protein "sensor" located on the surface of nerve cells that detects harmful stimuli and triggers the sensation of pain. It is expressed primarily in peripheral sensory neurons, the nerve cells that carry signals from the body to the spinal cord and brain, and functions as a polymodal detector of noxious heat, capsaicin, which is the compound responsible for the burning sensation of chili peppers, protons, osmotic stress, and various endogenous ligands, naturally occurring molecules produced by the body. Its central role in integrating painful stimuli and promoting nociception, the physiological process by which the nervous system detects and responds to potentially damaging stimuli, has made TRPV1 an ideal target for analgesic intervention. Experimental modulation of TRPV1 has demonstrated meaningful reductions in pain signaling in preclinical models through antagonism, blocking the receptor, or agonist-induced desensitization, using a stimulating molecule to exhaust and temporarily silence the receptor [1].

However, translating TRPV1-targeted strategies into safe and effective therapies presents significant challenges. Systemic TRPV1 blockade or activation, meaning treatment that affects TRPV1 receptors throughout the entire body, disrupts both pathological pain signaling and physiological thermosensation, the body's normal ability to sense temperature. Clinical and preclinical studies have shown that broad TRPV1 inhibition can produce dangerous thermoregulatory side effects, including elevations in core body temperature [2]. This risk arises because TRPV1 is sensitized by multiple converging stimuli, including protons and somatic changes, that collectively regulate channel gating, that is, whether the channel is open or closed [3]. As a result, nonspecific pharmacological approaches interfere with protective sensory functions alongside nociceptive signaling.

These safety concerns have constrained the clinical development of TRPV1-directed analgesics despite a strong biological rationale. The central challenge, therefore, is developing strategies that enable selective modulation of TRPV1 at sites of pathological pain while sparing receptors involved in systemic temperature regulation. Addressing this challenge requires delivery approaches capable of spatial and temporal precision, meaning the ability to deliver a drug to exactly the right location at the right time. Nanoparticle-engineered drug delivery systems have emerged as a promising solution. Nanoparticles are extremely small particles, typically between 1 and 1,000 nanometers in diameter, far smaller than a human hair, that can be engineered to carry and release therapeutic agents in a controlled manner. They are a well-established strategy for enhancing drug accumulation at target tissues while reducing systemic exposure, as demonstrated in fields such as oncology, including breast cancer targeting. The same principles can be applied to pain therapeutics to overcome the limitations of conventional TRPV1 modulation.

For TRPV1 targeting, nanoparticle-based systems offer three primary advantages. First, nanoparticles can localize therapeutic agents to TRPV1-expressing neurons, concentrating the active ligand where it is needed. Second, controlled release kinetics, the rate at which the drug is released over time, enable slow, sustained dosing, allowing desensitization without large peak concentrations that cause toxicity. Third, surface functionalization, whereby nanoparticles are chemically modified on their outer surface to seek out specific targets, enables receptor targeting. This can reduce off-target activation of related channels such as transient receptor potential ankyrin 1 (TRPA1) and thereby reduce irritation. The combination of localization and controlled release makes nanoparticles an attractive approach for avoiding thermoregulatory disruption while achieving lasting analgesia.

Despite growing interest, the literature on nanoparticle-enabled TRPV1 modulation remains highly diverse and fragmented. Studies differ widely in nanoparticle composition, size, surface charge, ligand functionalization, and stimulus-responsive release mechanisms. Outcomes are assessed using heterogeneous experimental models and metrics, making direct comparison difficult. In addition, the field spans multiple disciplines, further contributing to conceptual and methodological variability. These characteristics limit the utility of traditional systematic review approaches that rely on narrowly defined interventions and standardized outcome measures. A scoping review is the most appropriate methodological framework for synthesizing this body of work. Scoping reviews are designed to map the breadth of emerging or complex research fields, clarify key concepts, identify dominant design strategies, and highlight knowledge gaps without excluding studies based on design heterogeneity. This approach is well-suited to capturing both the mechanistic engineering features of nanoparticle systems and the broader research landscape shaping TRPV1-targeted nanotherapies. Additionally, it addresses bibliometric considerations, which are patterns in authorship and geographic output that could reveal potential disparities in methodological diversity and representation in existing work.

Accordingly, the objective of this scoping review is to analyze mechanistic and bibliometric trends in nanoparticle-engineered delivery systems designed to selectively modulate TRPV1 receptors for precision chronic pain therapy. The review addresses the following questions: (1) What nanoparticle design features are most commonly employed to achieve selective TRPV1 modulation? (2) How do characteristics such as particle size, surface charge, ligand functionalization, and stimulus-responsive release influence therapeutic selectivity and safety? (3) What bibliometric patterns characterize the research landscape, including influential publications, institutions, senior authors, and regions? The population of interest includes preclinical and translational models of chronic pain, the central concept is nanoparticle-mediated TRPV1 targeting, and the context encompasses nanoengineering approaches to pain management.

This scoping review aims to clarify how nanoparticle engineering is being leveraged to overcome long-standing barriers in TRPV1-targeted analgesia by systematically mapping interdisciplinary literature and identifying gaps that must be addressed to advance these technologies toward clinically viable chronic pain therapies.

## Review

Methods

Eligibility Criteria

Inclusion criteria required that studies within the top 100 most cited from Web of Science (WoS) address nanoparticle-based delivery systems in the context of TRPV1 modulation or related receptor pharmacology, and that they be published in peer-reviewed English-language journals, in order to facilitate cross-study comparison and consistency of analysis. Studies were excluded if they examined TRPV1 in non-pain contexts without pharmacological relevance, or were non-peer-reviewed sources such as conference abstracts or grey literature. No temporal limits were imposed, and publication years ranged from 2003 to 2024, capturing both foundational discoveries from the early 2000s and more recent advances in therapeutic research and drug development. 

Titles and abstracts of all retrieved records were independently screened against these predefined inclusion and exclusion criteria by a team of 8 trained interns. Full-text review was subsequently conducted for all records meeting initial screening criteria. Disagreements at any stage were resolved through discussion and consensus between reviewers. The full study selection process is summarized in the accompanying PRISMA Extension for Scoping Reviews (ScR) flow diagram in "Search Strategy."

Selection of Sources of Evidence

The WoS Core Collection was searched using relevant keywords. Retrieved results demonstrated a clear focus on TRPV1 in the context of inflammation and neuropathic modulation. To capture the most impactful research, the primary dataset was limited to the 100 most cited articles identified through the WoS Core Collection’s automation filter. Records and associated metadata were exported to Microsoft Excel (Microsoft® Corp., Redmond, WA) for subsequent analysis following database retrieval.

Information Sources

WoS was selected because it is a multidisciplinary database indexing high-impact journals across several scientific fields, including neuroscience, pharmacology, nanotechnology, and bioengineering. Access to this wide disciplinary range was essential for mapping the progression of TRPV1-targeted pain research over time, from early mechanistic discoveries to recent nanoengineering applications.

Search Strategy

A search strategy was developed using controlled keywords and Boolean operators to capture literature addressing TRPV1 signaling and nanoparticle-mediated drug delivery in the context of pain modulation. The keyword combination “TRPV1” OR “transient receptor potential vanilloid 1” AND “nanoparticle” AND “drug delivery” AND “chronic pain” OR “inflammatory pain” was applied within the WoS database. The search identified 108 records, with no exclusions performed by automated tools. The search was conducted on October 15, 2025.

**Figure 1:**
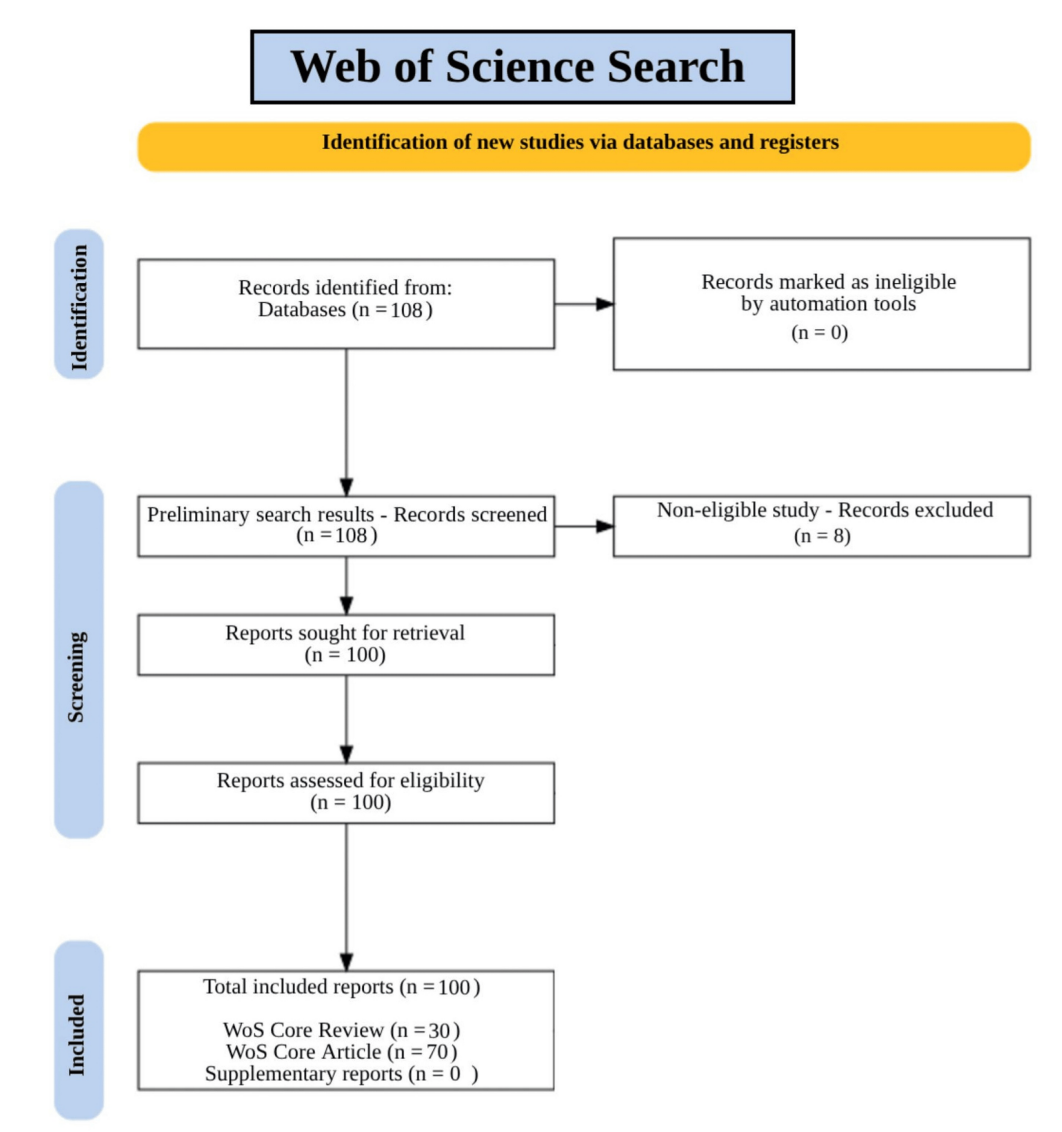
PRISMA Flow Diagram With Article Selection Process

Following screening, eight non-eligible records were removed, resulting in 100 included reports, consisting of 30 WoS Core reviews and 70 WoS Core research articles. The high inclusion rate indicated strong alignment between the search strategy and eligibility criteria. Inclusion of both review and original research articles strengthened the depth and contextual reliability of the evidence base. The selection process is depicted in a PRISMA 2018-compliant flow diagram in Figure 1 [4].

Several methodological limitations should be acknowledged. The search was conducted in WoS, and while this was selected to maximize coverage of biomedical and nanoscience literature, studies indexed exclusively in non-English or regional databases may not be represented, contributing to potential geographic selection bias consistent with the regional concentration observed in the results. Additionally, the citation-based component of the search strategy, used to identify high-impact records, may have introduced a citation circularity bias, wherein highly cited works are more likely to be retrieved and analyzed, potentially underrepresenting emerging or lower-resourced research groups. No formal risk-of-bias assessment tool was applied, as is consistent with scoping review methodology; however, variability in experimental design and outcome reporting across included studies limits direct cross-study comparability.

Data Charting Process

A standardized extraction template was used for data charting. Bibliometric and descriptive variables were recorded for each article, including first author, senior author, year of publication, journal title, total citation count in WoS, and primary research focus.

In addition to bibliometric variables, qualitative thematic data were charted to capture characteristics of nanoparticle design, including material composition, particle size, surface functionalization, TRPV1 modulation strategies, experimental models, and reported therapeutic outcomes. Consistent with scoping review methodology, data charting focused on identifying patterns and trends rather than quantitatively synthesizing effect sizes.

Synthesis of Results

Articles were summarized by identifying findings and themes relevant to the search keywords and research questions. Studies were subsequently grouped into thematic categories and qualitatively interpreted within the context of the review objectives.

Metadata were extracted and exported using Microsoft Excel and BibTeX (Oren Patashnik, Stanford University, Stanford, CA), and bibliometric trends were visualized in the R programming language (R Foundation for Statistical Computing, Vienna, Austria) using the Biblioshiny package (Massimo Aria and Corrado Cuccurullo, University of Naples Federico II, Naples, Italy). Bar plots illustrating key research trends were generated in Microsoft Excel. Conceptual illustrations were created using copyright-free NIH BioArt images (National Institutes of Health, Bethesda, MD) compiled with Kleki Paint Tool (Benjamin Vetter, Dresden, Germany) and Google Drawings (Google LLC, Mountain View, CA). Findings and methods were reported in compliance with the PRISMA 2018 guidelines for scoping reviews [5]. A meta-analysis was not conducted due to heterogeneity and the qualitative nature of the review.

Results

Bibliometric Analysis of Regional Authorship and Institutional Patterns

The most prominent thematic pathways included controlled agonist delivery for receptor desensitization and nanoparticle-mediated blood-brain barrier penetration, especially surface-functionalization strategies designed to enhance receptor specificity. These themes frequently co-occurred with capsaicin-based agonists and polymeric nanoparticle platforms, indicating a convergence toward precision TRPV1 modulation rather than broad receptor inhibition [6-10]. Across studies, engineering approaches increasingly emphasized localization and receptor-targeting ligands as core design principles for reducing systemic exposure while maintaining therapeutic activity.

**Figure 2:**
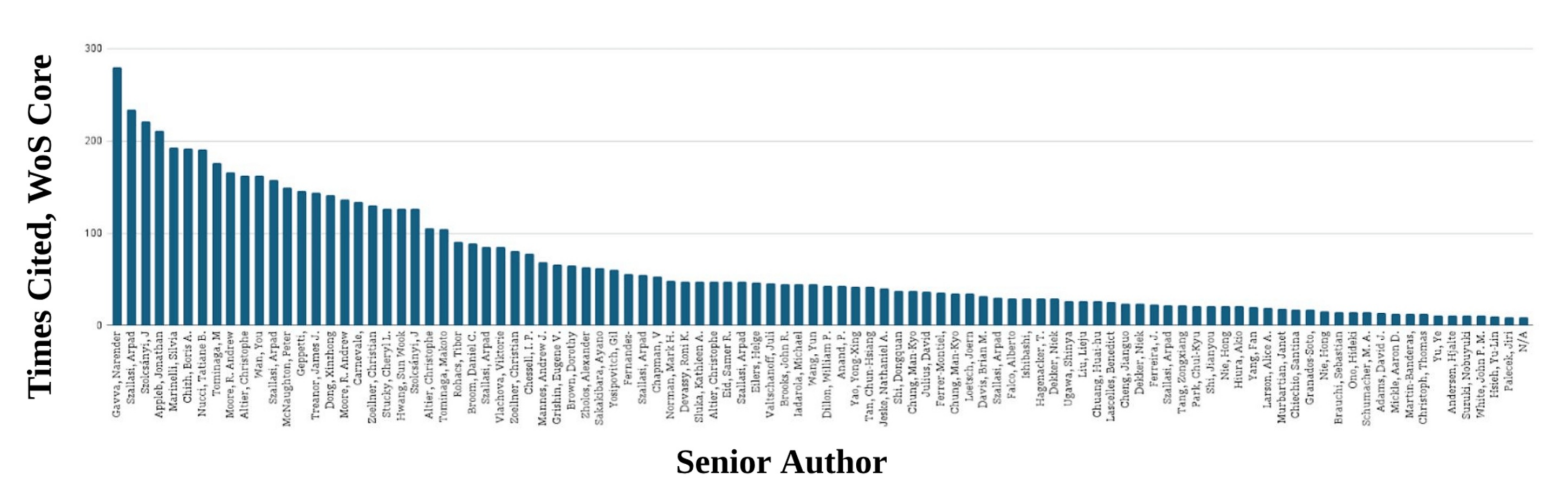
Senior Authors With Multiple Publications of the Top 100 Most Cited Studies, With Times Cited in Web of Science

Authorship analysis demonstrated a highly centralized citation structure, with a small cohort of senior investigators accounting for a disproportionately large share of influential publications. Citation patterns, reported in Figure 2, were concentrated among a limited number of leading researchers and institutions, a top-heavy distribution of research impact. Szallasi et al., Bölcskei et al., and Gavva et al. were associated with the largest number of highly cited keyword linkages (280, 234, and 221, respectively), reflecting strong contributions to TRPV1 signaling and pain modulation research [11,42,70]. After the top group of senior authors, there was a sharp decline in the number of investigators with multiple publications among the top 100 cited studies, further emphasizing the concentration of influence within a small research network. This concentration pattern is consistent with well-documented dynamics in bibliometric literature, wherein established investigators accumulate disproportionate citation advantages over time, a phenomenon sometimes described as cumulative advantage in scientific publishing.

**Figure 3:**
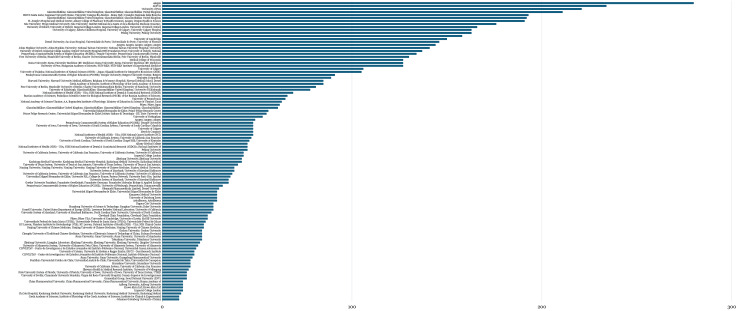
Senior Authors’ Institutions With Multiple Publications of the Top 100 Most Cited Studies, Times Cited in Web of Science (x) Plotted Against Institutions (y)

Institutionally, research output was dominated by resource-intensive academic and translational centers primarily located in the United States, Western Europe, and East Asia, as shown in Figure 3. These institutions frequently combined advanced nanofabrication capabilities with neurobiology and imaging infrastructure, enabling complex in vivo studies of nanoparticle biodistribution and receptor-level targeting. Amgen, AbbVie, and the University of Pécs contributed the largest number of publications, with Amgen producing the highest output by a substantial margin, showing institutional clustering analogous to the authorship distribution. Citation density within this dataset correlated with institutional characteristics, including access to multidisciplinary research teams and specialized infrastructure, consistent with patterns observed in capital-intensive biomedical research fields.

Regional analysis revealed distinct specialization patterns, with a small cluster of U.S., European, and East Asian research centers producing the majority of influential studies related to nanoparticle-based TRPV1 targeting and nanomedicine applications. This concentration shows the resource-intensive nature of the field. Consequently, smaller research institutions demonstrated lower representation, contributing to geographic concentration and potential reproducibility limitations across global research settings.

**Figure 4:**
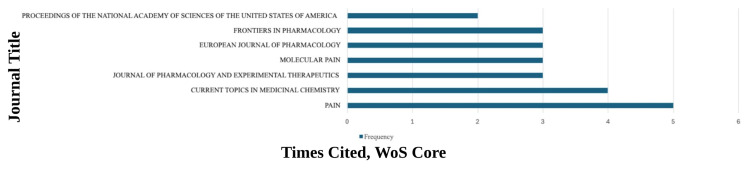
Journals With Multiple Publications of the Top 100 Most Cited Studies, With Times Cited in Web of Science

Journal-level distribution, reported in Figure 4, showed that Pain contained the highest number of publications among the top-cited records, followed by Current Topics in Medicinal Chemistry, while Journal of Pharmacology and Experimental Therapeutics, Molecular Pain, European Journal of Pharmacology, and Frontiers in Pharmacology contributed similar publication counts.

These patterns reveal a field shaped by a concentrated network of high-output investigators and institutions, with geographic and resource disparities that warrant consideration when interpreting the breadth and generalizability of the existing evidence base.

Mechanistic Findings on TRPV1 Modulation and Nanoparticle Design

TRPV1 agonists, most prominently capsaicin and capsaicinoid derivatives, were consistently employed to induce receptor activation followed by calcium-mediated desensitization, resulting in reduced nociceptive signaling [13-21]. Nanoparticle-encapsulated agonists demonstrated prolonged receptor engagement compared to free-drug formulations, with release kinetics influencing therapeutic outcomes across multiple investigated platforms [8,17,22-24]. Specifically, sustained low-dose release favored reversible TRPV1 desensitization, whereas rapid or high-dose exposure increased the likelihood of excitotoxic signaling and nociceptor ablation [8,17,23,24]. Calcium-mediated modulation was also observed in studies employing poly(ethylene glycol)-poly(lactic-co-glycolic acid) nanoparticles to enhance the effects of terpenes on pain pathways.

**Figure 5:**
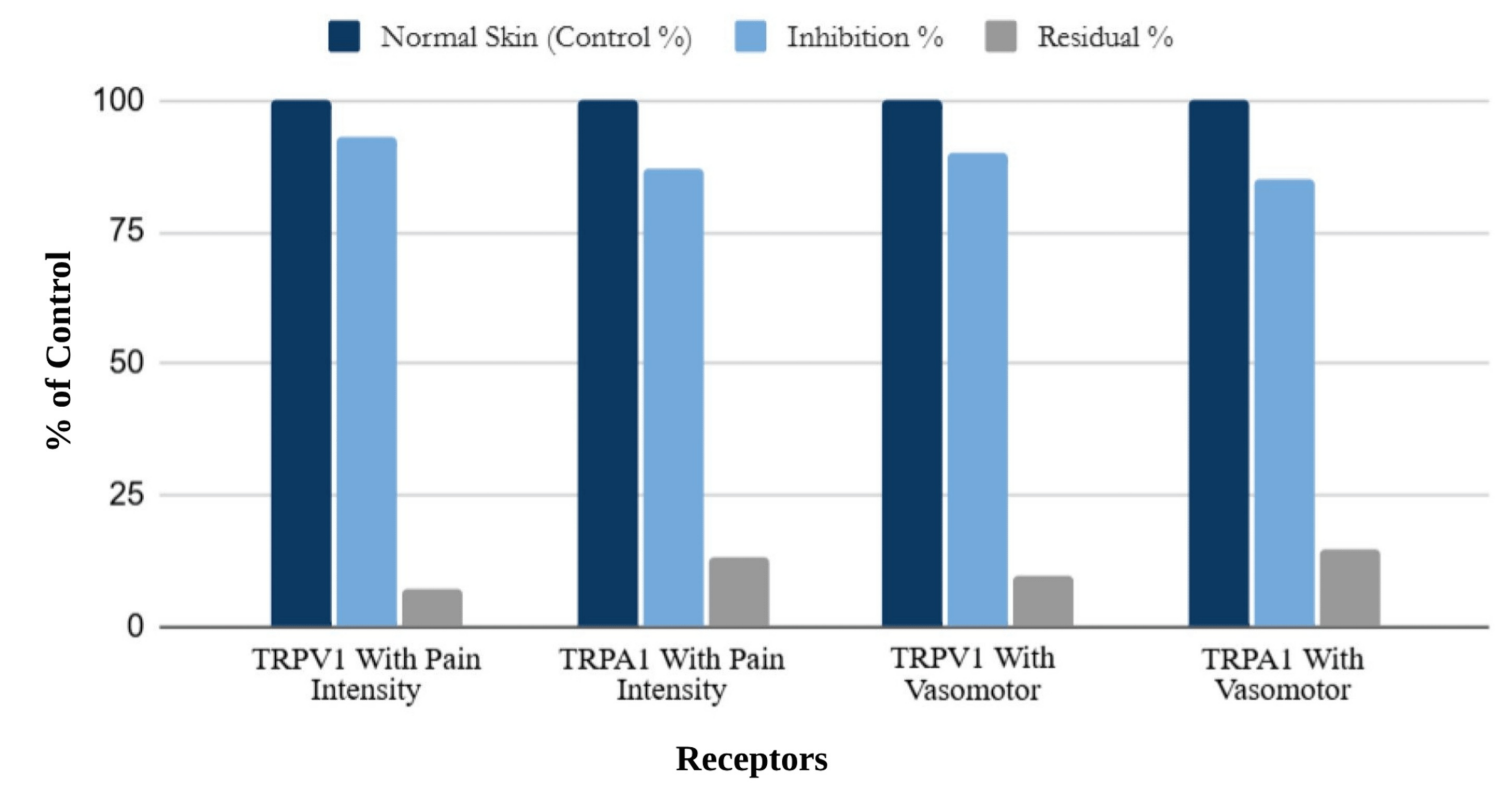
Ablation of Cutaneous Capsaicin-Sensitive Afferents and Inhibition Control of TRPV1- and TRPA1-Provoked Responses, Measured by Percent of Baseline Sensitivity

Capsaicin nanoparticle-mediated ablation markedly reduced both TRPV1- and TRPA1-mediated pain and vasomotor responses, with inhibition levels typically ranging from approximately 80% to 95% of control activity, as indicated in Figure 5. TRPV1 signaling consistently exhibited the greatest suppression and lowest residual activity, supporting the potential for strong analgesic outcomes when delivery systems effectively localize receptor modulation. Nanoparticle engineering directly influenced whether TRPV1 activation resulted in analgesia or nociceptor ablation, a distinction contingent on dosing profile and release rate [8,17,23,24].

Together, these findings emphasize that nanoparticle engineering parameters, particularly release rate and dose, are determinative of whether TRPV1 activation produces therapeutic desensitization or adverse nociceptor ablation.

Preclinical Successes and Translational Challenges

Temporal analysis demonstrated a gradual increase in TRPV1-focused nanoparticle research beginning in the early 2000s, followed by a pronounced expansion after 2019 that coincided with advances in nanofabrication and controlled drug delivery technologies. Highly cited publications were clustered within this later period, indicating increasing research output and impact as nanoparticle platforms matured.

Disease-specific categorization showed that most investigations focused on chronic neuropathic and inflammatory pain conditions, including osteoarthritis, sepsis-associated inflammation, peripheral neuropathy, and chemotherapy-induced neuropathic pain, with studies reporting varied but generally favorable outcomes across anti-inflammatory and analgesic endpoints [25-32]. In contrast, relatively fewer studies addressed visceral pain or central pain syndromes despite established TRPV1 involvement in these conditions, especially in migraines [11,33-36].

Within this context, keyword co-occurrence mapping demonstrated strong clustering among the terms "TRPV1," "capsaicin," "nanoparticles," "drug delivery," and "nociception," showing a dominant research emphasis on receptor modulation through engineered delivery systems. Emerging keywords such as "surface functionalization," "controlled release," and "blood-brain barrier" appeared more frequently in recent publications, indicating increasing technical sophistication over time [37-40].

Preclinical model analysis showed a strong reliance on animal-based assays, with most experimental studies employing swine or murine nociceptive pain models [41-44]. Human-derived sensory neuron models and advanced in vitro platforms were reported infrequently, and no clinical trials were represented among the top-cited records [33,45,46].

Across preclinical investigations, nanoparticle-mediated TRPV1 targeting consistently produced enhanced analgesic efficacy relative to free-drug controls. Compared with related ion channels such as TRPA1, TRPV2, and transient receptor potential melastatin 8 (TRPM8), TRPV1 remained the most consistently targeted channel for producing measurable analgesia in the included studies, while alternative channels were more frequently associated with inflammatory modulation or cold sensitivity pathways [47-55]. 

Capsaicin-based delivery systems showed the most consistent therapeutic performance among the evaluated TRPV1 modulators, while compounds such as SB-705498 and resiniferatoxin were investigated as alternative modulators, each presenting distinct challenges related to release control or nerve-ending ablation [56-61].

**Figure 6:**
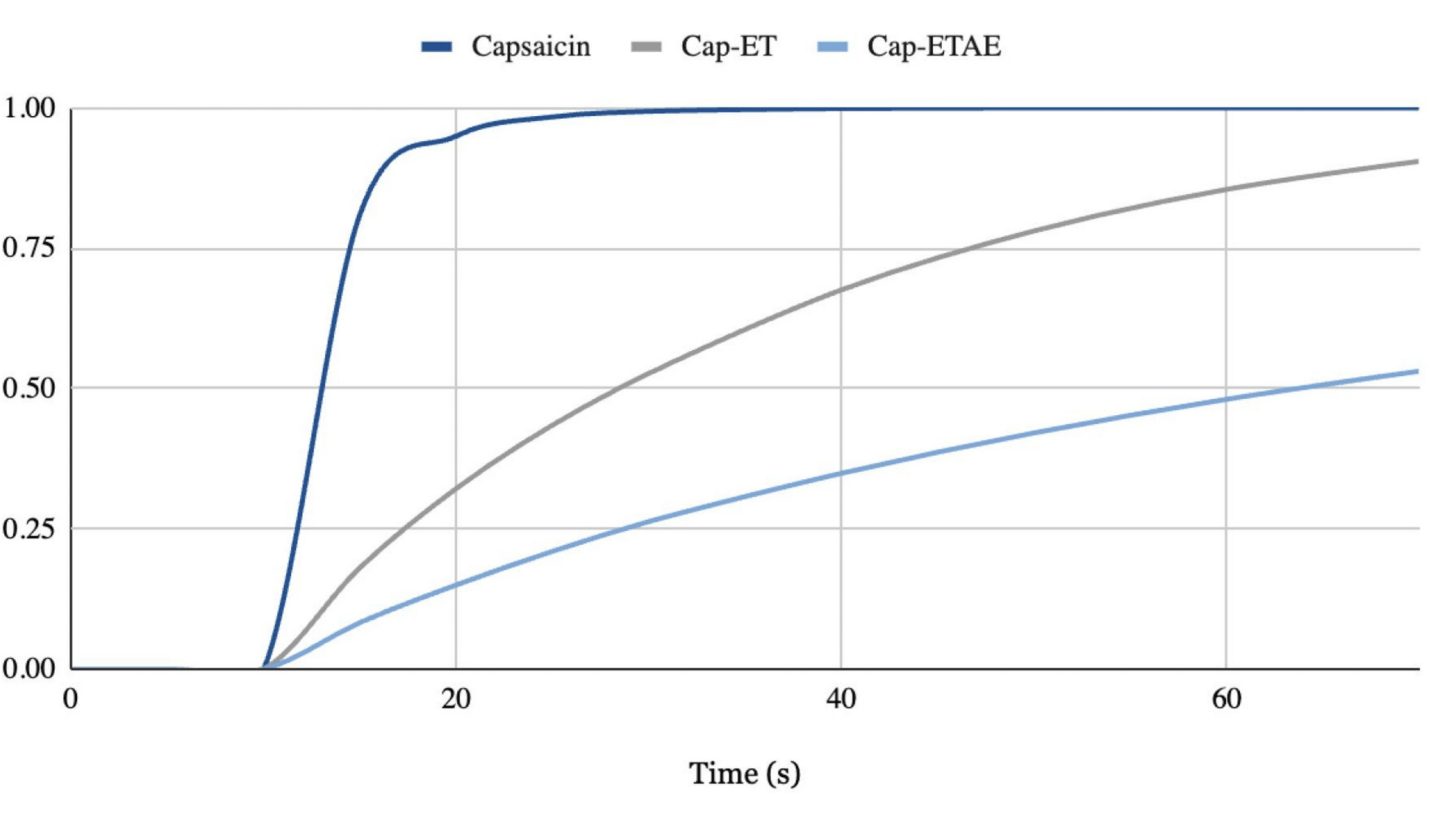
Relative Efficacy of Capsaicin vs. Charged Capsaicinoid

Beyond receptor-level modulation, TRPV1 expression in dorsal root ganglion neurons is closely linked to pain persistence, and pharmacological suppression of TRPV1 gene transcription has been associated with reductions in receptor levels and decreased responsiveness of associated transcription factors [62]. Among capsaicin, Cap-ET, and Cap-ETAE, capsaicin demonstrated the greatest peak efficacy, reaching a response of 1.00 by approximately 25 seconds, as indicated in Figure 6, whereas Cap-ET and Cap-ETAE exhibited more gradual and sustained modulation profiles more amenable to long-term chronic pain management [63].

Sustained-release nanoparticle formulations were associated with prolonged pain relief and reductions in neuroinflammatory markers, particularly when dosing profiles minimized peak exposure. It should be noted, however, that despite TRPV1's established involvement in itch pathways, receptor blockade has not demonstrated effectiveness in relieving this symptom in the reviewed studies [64].

**Figure 7:**
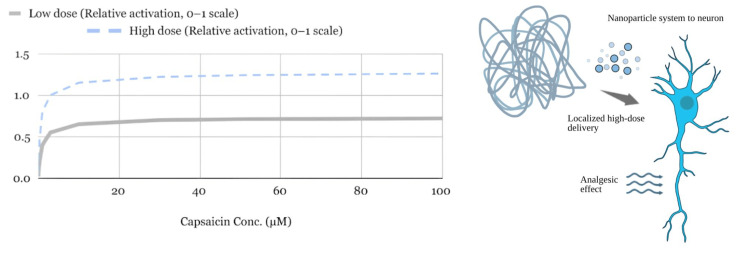
Controlled Capsaicin Nanoparticle Delivery (Low vs. High Doses) Neuron and nanoparticle image sources: NIH BioArt (Public Domain) (National Institutes of Health, Bethesda, MD) Editing tool: Kleki Paint Tool (Benjamin Vetter, Dresden, Germany)

Regarding dose-dependent effects, higher-dose controlled capsaicin nanoparticle delivery demonstrated approximately twice the reaction control compared to lower-dose formulations, as shown in Figure 7. While higher doses produced more pronounced analgesic effects at the TRPV1 receptor, they also carried greater potential for off-target activation, reinforcing the relevance of controlled release engineering to therapeutic optimization. 

Despite these preclinical successes, translational advancement remained limited, with studies frequently reporting variability in dosing thresholds and insufficient long-term safety data, collectively hindering progression toward clinical validation [3,65]. The synthesized literature also revealed several persistent gaps. Sex-specific biological responses to TRPV1 modulation and disease-tailored nanoparticle design strategies remain underinvestigated, and the absence of standardized outcome measures across experimental models limits cross-study reproducibility. The functional consequences of TRPV1 impairment at elevated temperatures, beyond hyperthermia induction, were rarely addressed in the included studies [66]. Few investigations systematically compared nanoparticle physicochemical and electrophysiological parameters across pain models, further constraining reproducibility [67-68].

Notably, several studies identified approaches capable of prolonging analgesia while mitigating thermoregulatory risk, including positive allosteric modulation targeting the outer pore of TRPV1 and structure-guided peptide engineering [2,21,69-74]. Scalability and manufacturing considerations were rarely addressed in the included literature, and regulatory pathways for nanoparticle-based TRPV1 therapies remained underexplored. These gaps reveal and emphasize the need for opportunities for future research, focused on clinically relevant delivery optimization, and broader geographic and demographic representation in preclinical study design.

While preclinical evidence consistently supports the analgesic potential of nanoparticle-mediated TRPV1 targeting, the absence of standardized dosing frameworks and long-term safety data is the critical barrier separating current experimental successes from clinically viable therapies.

Discussion

Scientometric Trends

Bibliometric analysis indicates that the intellectual development of TRPV1 pharmacology and nanoparticle-mediated delivery has been strongly shaped by a relatively small group of investigators whose early mechanistic discoveries established the experimental and translational framework still used in contemporary studies. Foundational work by Szallasi et al., Bölcskei et al., and Gavva et al. continues to influence receptor interpretation, assay design, and pharmacological benchmarking across the field [11,42,70]. Rather than reflecting only citation concentration, this continuity suggests that the field has evolved along a mechanistically anchored trajectory in which early discoveries regarding capsaicinoid signaling and TRPV1 thermoregulation remain central to ongoing nanoparticle engineering efforts. 

The geographic clustering of influential work within a limited set of U.S., European, and East Asian research centers likely reflects structural research capacity instead of purely academic visibility. Nanoparticle-based TRPV1 research requires integration of nanofabrication facilities and advanced imaging systems on top of heavy collaboration, creating high entry barriers for smaller institutions. Consequently, global representation in the literature remains uneven, which may restrict methodological diversity and slow the validation of delivery strategies across varied experimental environments.

Much research is centered around the idea of selective TRPV1 modulations, the use of electrophysiological and behavioral outcomes, and the importance of preventing inflammation or hyperactivation. This reflects the importance of modulating TRPV1 into an effective analgesia without any cognitive or motor side effects while acknowledging that TRPV1 hyperactivation leads to severe inflammation and weakening therapeutic benefits. However, capital-intensive infrastructure is required for blood-brain barrier and nanomedicine research. To overcome these limitations, there needs to be a stricter, more standardized, and common methodology to align channel-current modulation with behavioral analgesia and cognitive outcomes to enable transfer from in vitro to in vivo methods. 

Nanoparticle Efficacy in Translation

Precision-engineered nanoparticles optimized for size, surface charge, ligand density, and release kinetics have the potential to fundamentally reshape chronic pain therapeutics by selectively modulating TRPV1-expressing nociceptors and nanoparticles that can mitigate thermoregulatory disruption and prevent chronic pain signaling. Controlled receptor-specific targeting can further reduce negative side effects and toxic exposure, allowing efficient analgesia without damaging sensory functions. Across the literature, tissue penetration is shown to improve due to nanoscale size control, and surface charge allows for better circulation of nanoparticles, leading to immune evasion and off-target inflammation. Ligand density determines a receptor’s specificity, which is a major factor when designing pain medication using TRPV1 by regulating the amount of drugs and the speed of delivery in order to prevent any overstimulation and desensitization.

Despite strong preclinical efficacy signals, clinical translation of TRPV1-targeted nanoparticle therapies remains constrained by biological and pharmacological complexity. The receptor operates within a narrow therapeutic window in which small variations in exposure concentration or release kinetics can alter outcomes from reversible receptor desensitization to irreversible nociceptor damage [17,22,75]. While nanoparticle platforms improve pharmacokinetic control, the absence of consensus dosing frameworks makes it difficult to define universally safe therapeutic thresholds.

A second translational limitation arises from interspecies physiological differences. Rodent thermoregulation and TRPV1 expression patterns differ substantially from those observed in humans, reducing the predictive reliability of animal models for hyperthermic responses and long-term sensory effects [38,76-80]. Bridging this gap will likely require increased use of human-derived neuronal systems and hybrid translational models that better replicate human sensory physiology.

Comparative findings suggest that improvements in therapeutic performance are driven primarily by delivery engineering rather than by the discovery of fundamentally new TRPV1 ligands. Encapsulation of capsaicinoids and related agonists enhances ligand stability, prolongs tissue residence time, and reduces systemic exposure, collectively improving analgesic duration and tolerability [6,24,81-85]. These observations support the interpretation that delivery strategy has become the dominant determinant of functional outcomes in TRPV1-directed therapies.

**Figure 8:**
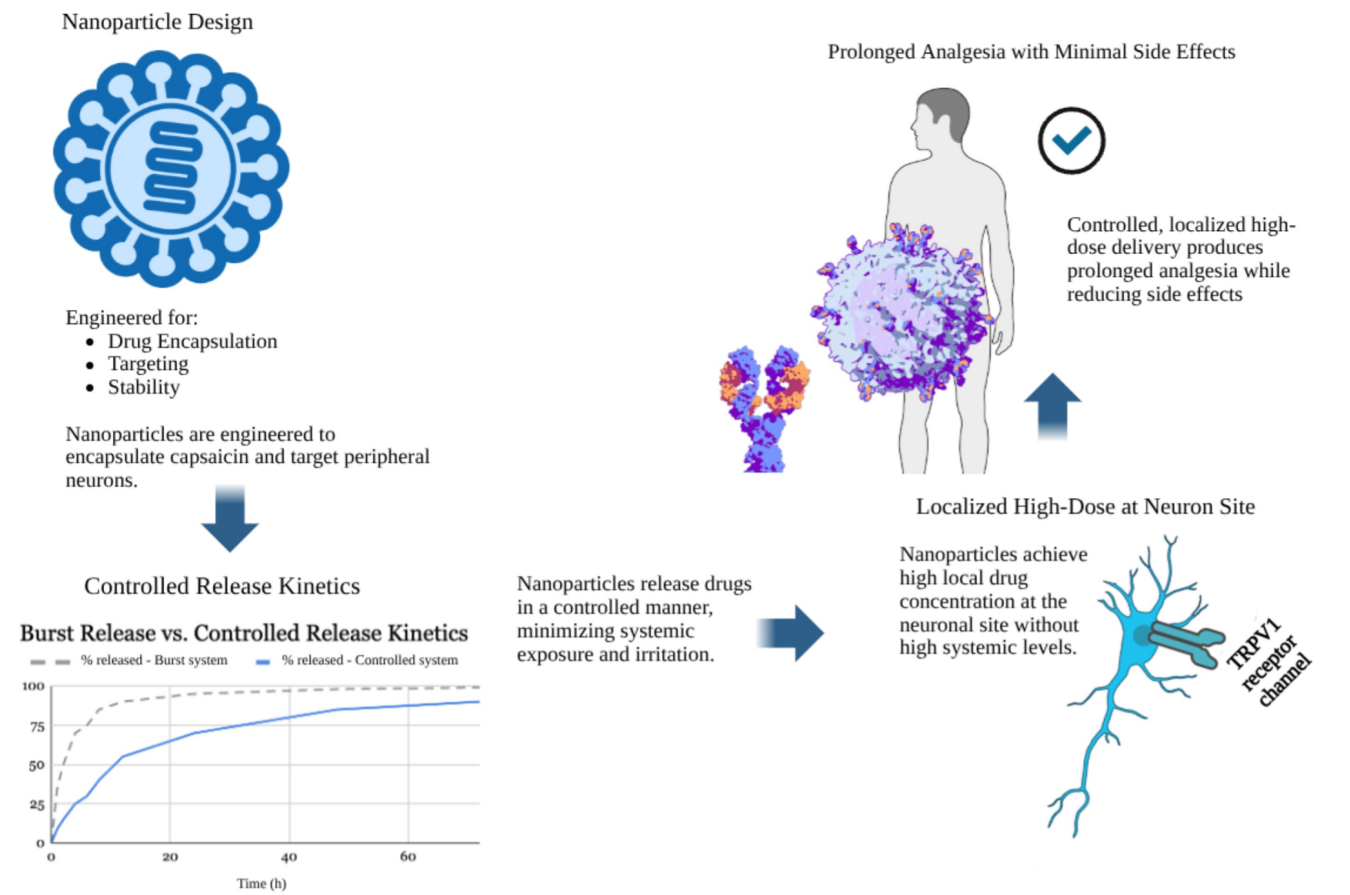
The Flow of a Nanoparticle-Engineered Dose Modulation System Nanoparticle design, neuron, receptor, and analgesia images: NIH BioArt (Public Domain) (National Institutes of Health, Bethesda, MD) Editing tool: Kleki Paint Tool (Benjamin Vetter, Dresden, Germany)

However, effectiveness varies across nanoparticle classes. Polymeric and surface-functionalized platforms generally provide more predictable release kinetics and receptor targeting than lipid-only carriers, while magnetic and stimuli-responsive systems offer improved spatial precision at the cost of increased manufacturing complexity and scalability challenges [2,86-88]. Future optimization efforts will therefore need to balance mechanistic precision with production feasibility to ensure translational viability in the flow of a nanoparticle-engineered dose modulation system, documented in Figure 8.

Interpretative Constraints

Reproducibility remains a major interpretative constraint in TRPV1 nanoparticle research due to variability in experimental design, nanoparticle synthesis protocols, and outcome definitions. Differences in particle size distributions, surface charge characteristics, ligand density, and release mechanisms limit direct cross-study comparisons and complicate attempts at quantitative synthesis [89-93]. Without standardized characterization and reporting practices, incremental engineering improvements are difficult to benchmark across independent investigations.

Interpretive uncertainty is further amplified by inconsistent definitions of analgesic success, with some studies emphasizing behavioral endpoints while others prioritize molecular or electrophysiological measures [46,55,65,94-102]. Establishing shared reporting standards, unified outcome metrics, and reproducible nanoparticle characterization frameworks will be essential steps for advancing TRPV1-targeted nanotherapies toward clinically meaningful validation.

## Conclusions

TRPV1-targeted nanoparticles show strong potential, but in order to implement these ideas into clinical therapies, they will require access to infrastructures, reproducibility, and standardized methodologies. Future research should prioritize developing an adaptive nanoparticle system that includes stop mechanisms, rescue ligands, and release triggers to determine quick solutions in cases of hyperactivation. To ensure long-term use and safety of a drug, researchers need to study how the human brain and body will react to the new analgesia over long periods of time.

The integration of TRPV1 receptor biology with nanomedicine has the potential to transform chronic pain treatment in the future. By avoiding thermoregulatory complications, nanoparticle delivery systems can finally make TRPV1 modulators clinically viable. If successful, this approach could reduce dependence on opioids and improve quality of life for chronic pain patients and expand treatment options for conditions resistant to traditional drugs.
